# VAP: a versatile aggregate profiler for efficient genome-wide data representation and discovery

**DOI:** 10.1093/nar/gku302

**Published:** 2014-04-21

**Authors:** Charles Coulombe, Christian Poitras, Alexei Nordell-Markovits, Mylène Brunelle, Marc-André Lavoie, François Robert, Pierre-Étienne Jacques

**Affiliations:** 1 Département d’informatique, Faculté des sciences, Université de Sherbrooke, Sherbrooke, Québec, J1K 2R1, Canada; 2 Institut de recherches cliniques de Montréal, Montréal, Québec, H2W 1R7, Canada; 3 Département de biologie, Faculté des sciences, Université de Sherbrooke, Sherbrooke, Québec, J1K 2R1, Canada; 4 Département de médecine, Faculté de médecine, Université de Montréal, Montréal, Québec, H3T 1J4, Canada; 5 Centre de recherche du Centre hospitalier universitaire de Sherbrooke, Sherbrooke, Québec, J1H 5N4, Canada

## Abstract

The analysis of genomic data such as ChIP-Seq usually involves representing the signal intensity level over genes or other genetic features. This is often illustrated as a curve (representing the aggregate profile of a group of genes) or as a heatmap (representing individual genes). However, no specific resource dedicated to easily generating such profiles is currently available. We therefore built the versatile aggregate profiler (VAP), designed to be used by experimental and computational biologists to generate profiles of genomic datasets over groups of regions of interest, using either an absolute or a relative method. Graphical representation of the results is automatically generated, and subgrouping can be performed easily, based on the orientation of the flanking annotations. The outputs include statistical measures to facilitate comparisons between groups or datasets. We show that, through its intuitive design and flexibility, VAP can help avoid misinterpretations of genomics data. VAP is highly efficient and designed to run on laptop computers by using a memory footprint control, but can also be easily compiled and run on servers. VAP is accessible at http://lab-jacques.recherche.usherbrooke.ca/vap/.

## INTRODUCTION

Genomic data are often represented over genes or other regions of interest as aggregates or as individual profiles showing the spatial distribution of the signal intensity. Such representations are particularly useful for interpreting spatial or intensity variations of the signal between experimental conditions (1–12). However, the absence of a general stand-alone tool that allows for easy customization of such representations forces most laboratories to develop their own in-house script. Some stand-alone tools such as CEAS (13) also integrated into Cistrome (14), ACT (15) and seqMINER (16) do offer aggregate profiles in their outputs. However, these tools do not allow users to provide the coordinates of their regions of interest or modify parameters such as the resolution and the number of reference points. Moreover, and most importantly, these tools mainly use a constant number of windows (relative method) to represent genes and their flanking intergenic regions. As demonstrated below, the relative method should be used with caution and an alternative method, using constant window size and thus termed absolute method, represents a better approach. We therefore developed versatile aggregate profiler (VAP), a stand-alone intuitive tool designed to analyze very high volumes of experimental data on laptop computers, and which supports both the absolute and relative methods. Based on a simple gene list, VAP generates aggregate or individual graphs of the genomic signal using a customizable number of windows over a specified number of reference points. These reference points delimit the genes of interest as well as their flanking genes, or even exons. Alternatively, VAP can directly use genomic coordinates defined by the user (e.g. transcription factor binding sites). The output files include values such as standard error of the mean (SEM) to facilitate statistical comparisons between groups of features or datasets. VAP is accessible through both a user-friendly platform-independent Java interface or via command line to provide flexibility to advanced users. 

## RESULTS

### The importance of using windows of constant length

**Figure 1.**
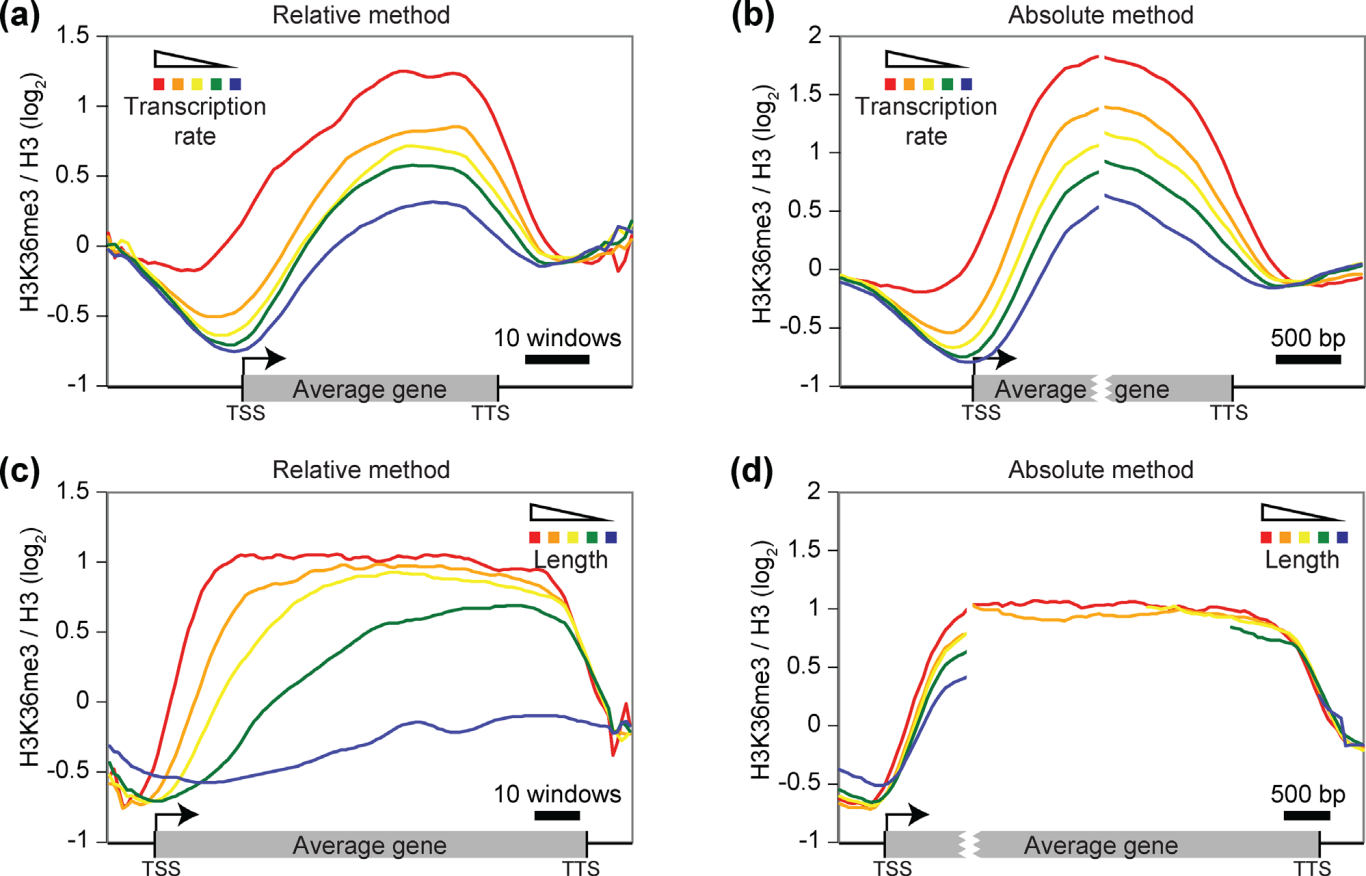
Comparison of the relative and absolute methods. When genes are grouped based on criteria other than length, the relative and absolute methods gave similar results. (**a–b**) Aggregate profiles showing H3K36me3 (17) on groups of genes based on their transcriptional frequency (26) using the annotation mode and either the relative (a) or the absolute (b) method. The five groups from red to blue contain, respectively, 231 genes transcribed at a rate above 16 mRNA/h, 1053 genes with a rate between 4 and 16 mRNA/h, 955 genes between 2 and 4 mRNA/h, 1045 genes between 1 and 2 mRNA/h and 1492 genes with a rate below 1 mRNA/h. (**c–d**) Aggregate profiles showing the same dataset as in panels a–b on groups of genes based on their length using the relative (c) or the absolute (d) method. The five groups from red to blue contain, respectively, 84 genes longer than 5 kb, 405 genes with a length between 3 and 5 kb, 801 genes between 2 and 3 kb, 2227 genes between 1 and 2 kb and 3087 genes with a length of less than 1 kb.

In the first aggregate representations of Chromatin Immunoprecipitation (ChIP) experiments hybridized on tiling arrays, all genes were divided in a constant number of 40 windows (17). This method, easy to reproduce, was then used by many groups (18–24) and included in recently developed web tools such as the ‘Gene plot’ section of the WashU Epigenome Browser (25). As a consequence of this methodology, the size of the windows varies according to gene length. For instance, the size of each window for a 400 bp-long gene divided into 40 bins is 10 bp, while the window size is 100 bp for a 4 kb-long gene. In the seminal publication by the Young group (17), and as reproduced in Figure 1a, the genes from *Saccaromyces cerevisiae* were grouped based on their transcription frequency and the graph shows that the level of the histone modification H3K36me3 correlates positively with the level of transcription.

Considering that, in the above approach, all genes were divided into the same number of windows then, for example, the signal in the fourth bin (out of 40) would represent the signal at 10% of the gene length; we therefore name this approach the ‘relative’ method. However, it is unlikely that the transcriptional machinery would be able to sense the relative distance from the transcription start site (TSS). To circumvent this conceptual problem, we have previously proposed (1) that genes be divided into windows of constant size, thereby using a varying number of windows for genes of different lengths. We have named this approach the ‘absolute’ method and have employed it in multiple studies (1–12). As illustrated in several examples below, representing genomic data using the absolute method appears to better reflect biological evidence (27). To generate aggregate profiles using the absolute method, one has to determine the number of windows to represent the average feature. In cases where both the start and end coordinates of genes are used as reference points (anchors) to align the signal, this produces an interruption in the profiles for genes having a length different from the represented length (number of windows times window size). For instance, in Figure 1b, all genes were virtually cut in the middle of the gene, and the signal aligned at both ends.

Considering that each group contains a mix of genes of different lengths, both the relative and absolute methods produce similar aggregate profiles (Figure 1a and b). However, if genes are grouped based on their length (each group containing a mix of transcriptional levels), the methodology has a significant impact on the output (Figure 1c and d). This is due to the fact that, using relative method, the gene length influences the window size. Consequently, a signal appearing at the same distance from the TSS (e.g. 200 bp) for a long and a short gene will be placed in different windows (e.g. first window of a 4 kb gene compared to the 20th window of a 400 bp gene divided into 40 windows). Without taking this bias into account, one could incorrectly interpret Figure 1c as showing that H3K36me3 accumulation rate correlates with gene length. In striking contrast, based on the absolute method, and by aligning the first kb of the genes before the split, it is clear that gene length has no impact on the accumulation rate of H3K36me3. Rather, H3K36 trimethylation accumulates as a function of the distance from the TSS at a rate that does not differ between long and short genes (28).

### Versatile functionalities

**Figure 2.**
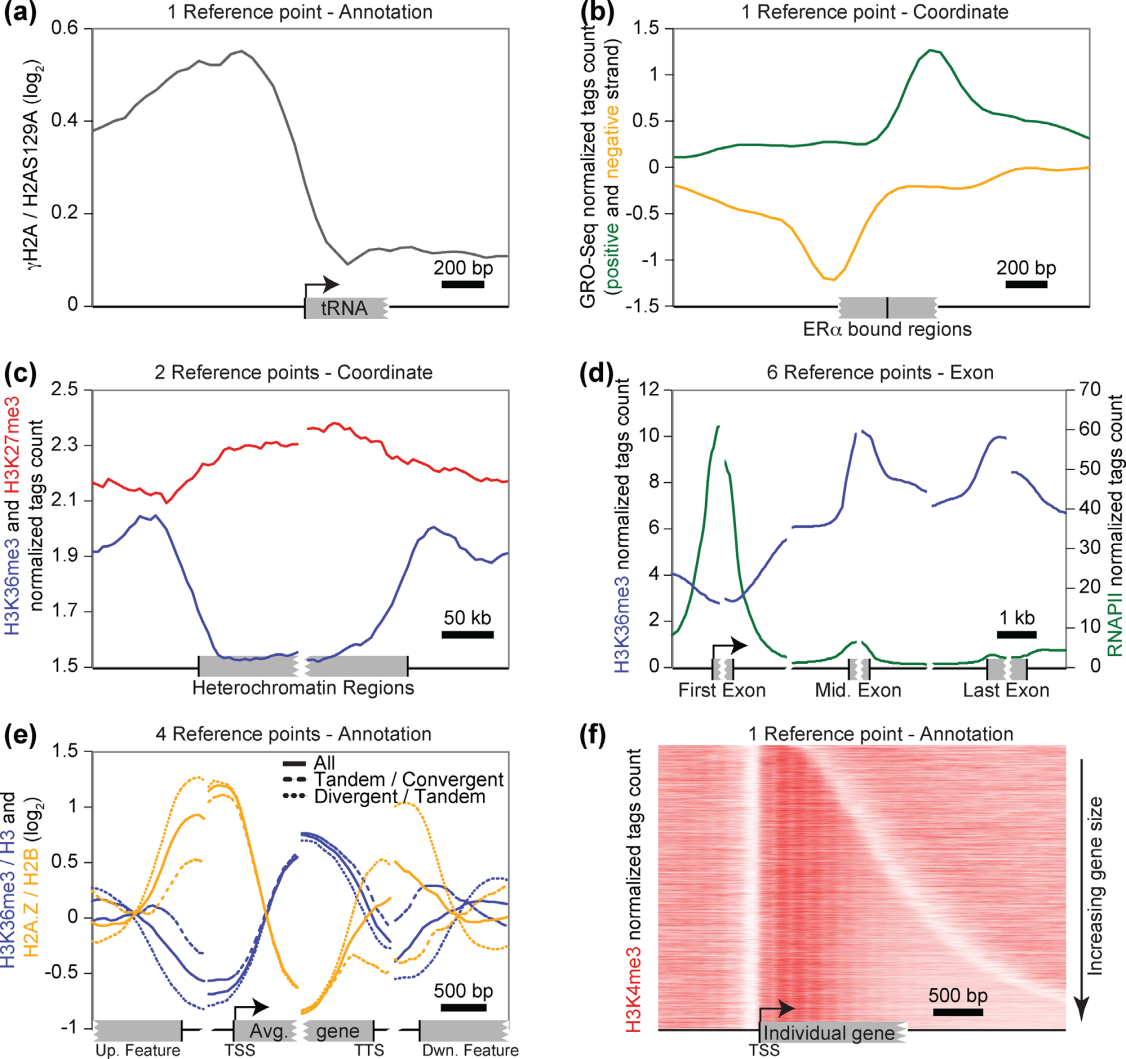
The versatile functionalities of VAP. Many options are offered to users such as three analysis modes (annotation, coordinate and exon) using one to six reference points, with the possibility of automatically subgrouping annotations based on the orientation of adjacent annotations. The black vertical bars on the X-axis represent the position of the reference point(s). (**a**) Aggregate profiles, generated using the annotation mode and one reference point, showing the γH2AS129p ChIP-chip dataset (2) in yeast cells over a group of 275 non-mitochondrial tRNA genes. (**b**) Aggregate profiles, generated using the coordinates mode and one reference point, showing a GRO-Seq dataset (GSE45822) in MCF7 cells (after 40 min E2 stimulus) (31) over a group of ERalpha-bound potential active enhancers based on the co-enrichment of ERalpha (E-TABM-828) (32) and H3K27ac (GSM945854) (33) by ChIP-Seq in MCF7 cells. The reads from the GRO-Seq experiment mapping to the negative strand were assigned a negative score. ERalpha summits were identified using MACS (34) then filtered to keep only those in intergenic regions and located at more than 3 kb of known TSS. The H3K27ac signal was then calculated over a 1 kb window centered on ERalpha summit and a threshold applied. (**c**) Aggregate profiles, generated using the coordinate mode and two reference points, showing the H3K36me3 (blue) and H3K27me3 (red) ChIP-chip datasets from ENCODE (33) in U2OS cells over a group of 30 heterochromatin regions from chr19 (3). (**d**) Aggregate profiles, generated using the exon mode and six reference points, showing the H3K36me3 (blue) and RNAPII (green) ChIP-Seq datasets from ENCODE (33) in HeLa cells over the exons of the 44,202 refSeq human genes. (**e**) Aggregate profiles, generated using the annotation mode and four reference points, showing the H3K36me3 (17) (blue) and H2A.Z (35) (orange) ChIP-chip datasets in yeast cells over a group containing the 6576 non-mitochondrial genes from sacCer1 (plain curves), as well as on subgroups of genes based on the orientation of the adjacent annotations. Only the 1637 upstream tandem and downstream convergent genes (dashed curves) and the 1588 upstream divergent and downstream tandem genes (dotted curves) subgroups are shown. (**f**) Individual profiles showing the H3K4me3 MNase-ChIP-Seq dataset (GSM1016879) in yeast cells over the 4259 genes without missing data of the sacCer3 assembly (sorted by their length), generated using the annotation mode and one reference point. The heatmap representation was performed using TreeView (http://rana.lbl.gov/EisenSoftware.htm). Both the upstream and downstream nucleosome-free regions (represented by the white bands) are striking, as are the 4–5 first nucleosomes immediately downstream of TSS.

VAP offers various functionalities from an intuitive interface. The most common usage is to generate aggregate profiles of signal along genes aligned at both their start and end boundaries, requiring two reference points as shown in Figure 1. To generate these profiles over five groups of genes, five files are required, each simply a compilation of gene names. The genomic coordinates of each reference point are extracted from a genome annotations file using the gene name as the key. Three types of files are therefore required in this analysis mode called ‘annotation’: the files containing the gene names (called the reference groups), a genome annotations file and the dataset files containing the (usually normalized) signal to be analyzed. As an option, selection and exclusion filters can be dynamically applied to the reference groups (e.g. genes grouped by transcription rate onto which a filter on gene length is applied). It is crucial for the genome annotations file and the datasets to be from the same assembly to avoid potential shifts in the representation. Reference groups can contain types of genetic features other than protein-coding genes, as long as they are included in the genome annotation file (Figure 2a). VAP can also be used in the analysis mode, called ‘coordinate’, where the reference group files contain the genomic coordinates provided by the user thus making the genome annotations file unnecessary. This provides users with the flexibility of mapping their data onto any genomic region, such as profiling transcriptomic data over binding sites identified in a ChIP-Seq experiment (Figure 2b). As for the annotation mode, the coordinate mode can be used to generate aggregate profiles on one or more reference points, and the orientation of the regions is also taken into consideration (Figure 2c). VAP supports up to six reference points, which are used in the last analysis mode called ‘exon’ where aggregate profiles are generated independently on the first, middle and last exons (Figure 2d). Using the exon mode, one can rapidly determine that H3K36me3 is enriched over exons relative to introns, as previously reported (29,30). Such local enrichment in exons cannot be detected by looking at the same datasets using the annotation mode and only two reference points (Supplementary Figure S1a). Aggregate data are usually displayed as the average signal of the reference group (with the possibility of displaying standard deviation (SD) and SEM) but VAP can also output median, maximum and minimum aggregate values. All of these analyses were conducted with a window size (resolution) of 50 bp and a smoothing of six sliding windows applied on the aggregate data, but these parameters are also customizable.

For compact genomes such as yeast, where the distance between genes is ∼500 bp, it is important to delimit the intergenic regions in order to avoid signal contamination from adjacent genes. This can be done using four reference points (two on each side of the intergenic regions flanking the genes of interest). As illustrated in Figure 2e (plain curves), the histone modification H3K36me3 (blue) is clearly restricted to genes while the histone variant H2A.Z (orange) is clearly restricted to intergenic regions. This observation is not as clear when using only two reference points (Supplementary Figure S1b) and is even clearer when using six reference points to delimit the boundaries of the flanking genes (Supplementary Figure S1c). Another important aspect to take into account when working with compact genomes is the impact of the orientation of adjacent genes. This is particularly well illustrated using the case of H2A.Z (Figure 2e). When considering all genes without respect to the orientation of their neighbors, H2A.Z appears to be enriched in both the upstream and the downstream intergenic regions (Figure 2e, plain orange). However, the enrichment in the downstream intergenic region is lost when only the subgroup of genes having a downstream neighbor in the convergent (tail–tail) orientation are considered (Figure 2e, dashed orange) and it increases for the subgroup of downstream genes in the tandem (tail–head) orientation (Figure 2e, dotted orange). This asymmetry is also apparent in the upstream intergenic region by comparing the divergent (head–head) genes (Figure 2e, dotted orange) and the tandem genes (Figure 2e, dashed orange). Based on this easy to use function of VAP, one can quickly conclude that H2A.Z is enriched in the upstream intergenic regions (promoters), but absent (or present at much lower levels) in the downstream intergenic regions (terminators).

In addition to aggregate profiles, VAP can also output individual profiles that can then be used for heatmap representation and/or clustering analyses (Figure 2f). This representation has the advantage of adding an extra dimension to the data by, for example, sorting genes based on gene length, transcription rate or other properties.

### The impact of the methodology

**Figure 3.**
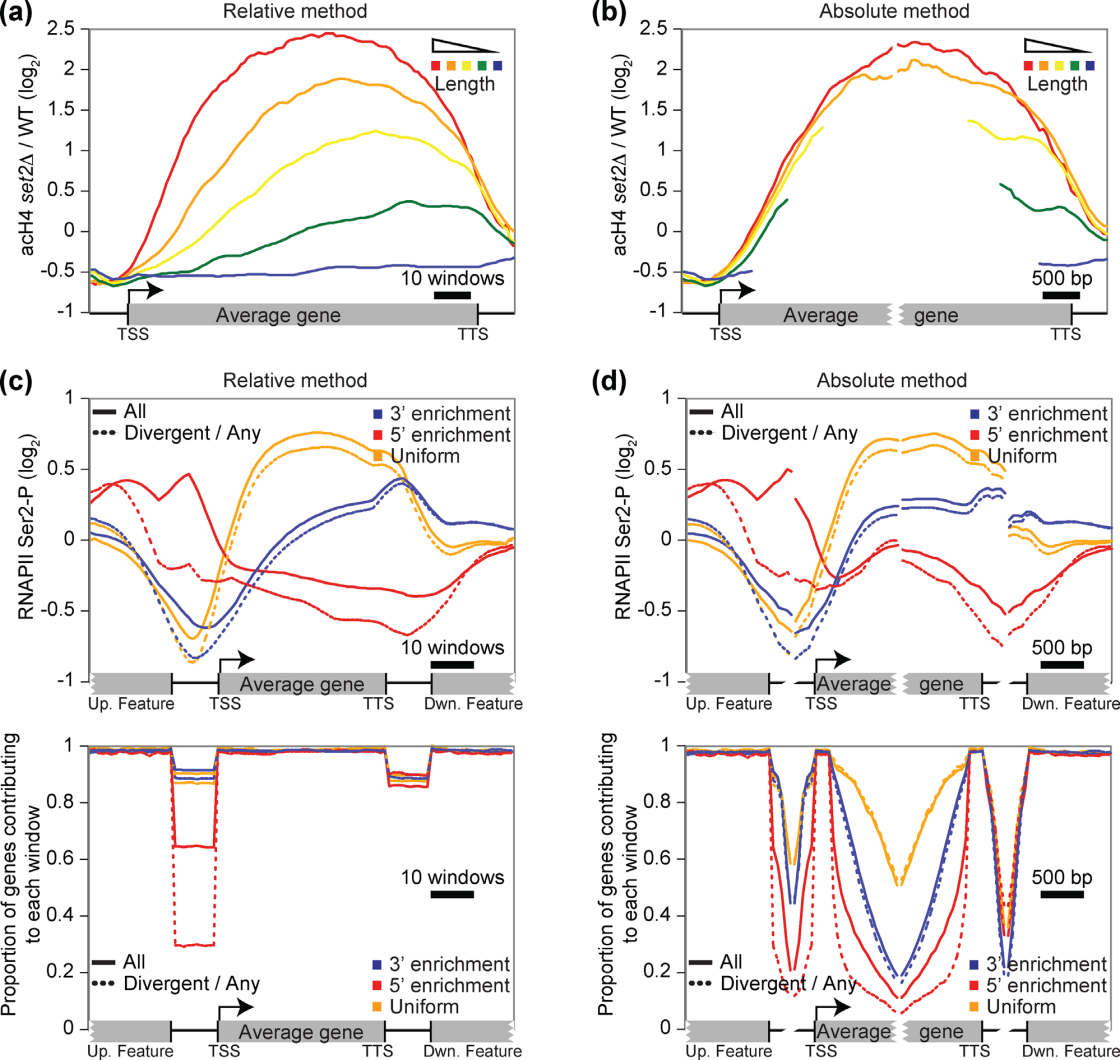
A biased methodology can lead to erroneous conclusions. Using the relative method to analyze genes grouped by their length is not appropriate and considering the orientation of proximal genes is crucial for compact genomes. (**a–b**) Aggregate profiles showing the acH4 dataset difference from a strain deleted for the *SET2* gene and the corresponding wild-type strain (18) on groups of genes based on their length using the relative (a) or the absolute (b) method. The groups of genes used are the same as in Figure 1c and d. (**c–d**) Aggregate (top) and proportion (bottom) profiles showing the RNAPII Ser2p dataset (19) on groups of genes identified to show the ‘normal’ 3’ enrichment (blue), an unusual 5’ enrichment (red), and an unusual uniform profile (orange) from the complete groups (plain curves) and the subgroup of genes in the divergent orientation with the upstream gene (dashed curves) using the relative (c) or the absolute (d) method. Each group contains, respectively, 3806, 723 and 863 genes, and the subgroups 2112, 313 and 506 genes.

It was originally reported that long genes depend on the Set2/Rpd3S pathway for accurate transcription (18). This conclusion arose from using the relative methodology to analyze genes grouped by their length. As reproduced here in Figure 3a, the authors compared the impact of deleting the *SET2* gene on histone H4 acetylation levels. Based on this graphical representation, it is tempting to conclude that ‘deletion of *SET2* led to a more dramatic increase in acetylation at genes with longer Open Reading Frames (ORF), suggesting that Set2 dependence was proportional to gene length’ (18). However, using the absolute method (Figure 3b), and as refuted by others (36), one would conclude that gene length has no significant impact on the role of Set2 in the dynamics of histone H4 acetylation.

In another case, a group studying the phosphorylation of the RNA polymerase II (RNAPII) C-terminal domain identified ‘gene class-specific patterns’ (19). As illustrated in Figure 3c (top) by the plain blue curve, the level of Ser2 phosphorylation (Ser2p) gradually increases toward the 3’ end of genes, as generally accepted (27,37–38). In their study, Tietjen *et al.* (19) also identified two groups of genes with unusual Ser2p profiles. In the first group, Ser2p peaks at the 5’ of the genes (Figure 3c, top, plain red curve), while in the second group, Ser2p is uniformly distributed over the length of the genes (Figure 3c, top, plain orange curve). To identify these unusual groups, the authors used the relative methodology and generated individual profiles that were later submitted to hierarchical clustering. However, using the absolute method, and as reported by others (9,39), the same data suggests rather that all groups have the same gradual accumulation of Ser2p toward the 3’ end of genes (Figure 3d, top). Furthermore, using the automatic subgrouping functionality of VAP to display the subgroup of genes divergent to the upstream gene (head–head), it appears that the unusual accumulation of Ser2p in the upstream intergenic region greatly decreases (Figure 3c and d, top, dotted red curve). Also, and as acknowledge by the authors (19), the genes with apparent uniform Ser2p distributions are enriched for highly transcribed genes, while the genes enriched at the 5’ are in general less transcribed than the genes with the normal 3’ enrichment (Supplementary Figure S2a, note the SEM), explaining the difference in the maximal Ser2p accumulation between the three groups.

In addition to the aggregate signal profiles (Figure 3c and d, top), VAP also generates a graph containing, for each window of each group, the proportion of the group members contributing to the aggregate profile in the corresponding window (Figure 3c and d, bottom). Looking at this graph generated using the relative method, it is quite striking that approximately 35% of all genes with a 5’ Ser2p enrichment and approximately 65% of the subgroup with divergent upstream orientations actually overlap with the upstream gene (therefore not contributing to the signal in the upstream intergenic region) while this is the case for only approximately 10% for the other groups of genes (Figure 3c, bottom). This overlap clearly contributes to the accumulation of Ser2p signal in the 5’ of these genes. Moreover, the proportion graph from the absolute method clearly illustrates that genes with uniform Ser2p distribution are on average longer than the genes with the canonical 3’ enrichment, while the genes with the 5’ enrichment are shorter (Figure 3d, bottom). As shown in Figure 1, this length difference also contributes to explaining the profiles obtained using the relative method. Analyzing the data with VAP allows one to quickly detect that the genes in these groups actually possess unusual properties that together explain their profiles. Taken together, these two examples demonstrate that the choice of methodology to represent the data can have an important impact on the biological interpretation.

### VAP is designed to run on either laptop computers or servers

**Figure 4.**
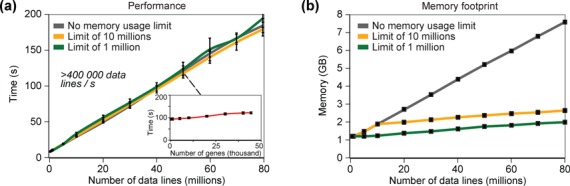
VAP is efficient and designed to minimize the memory footprint. The computing time and memory footprint is linear to the number of data lines in the dataset, except for the memory footprint that can be limited, but is almost invariant to the number of annotations to analyze. The tests were conducted on a 2.2 GHz computer with a SATA hard drive. (**a**) Computing time to process the H3K36me3 ChIP-Seq datasets (33) (used in Figure 2d) downsized and profiled on a group containing 44,202 refSeq genes of the human genome (extracted from the UCSC genome browser (40)) without (grey) or with a limit of 10 million (orange) or 1 million (green) data lines read at a time. The file containing 50 million lines was used to calculate the computing time of varying numbers of genes in the reference group (inset). The SD of 10 replicates are shown. (**b**) Memory footprint to process the same datasets as panel a. The SD were too small to be shown.

The performance of VAP is linear to the number of lines in the dataset, such that a dataset of 50 million lines in BedGraph format is processed in about 2 min on a group containing all the genes in the human genome (Figure 4a, grey curve). This performance of more than 400 000 lines per second is almost invariable to the number of annotations (genes) to be analyzed (Figure 4a, inset). The user can also minimize the memory footprint of VAP (Figure 4b) without affecting performance (Figure 4a). Performance will be eventually improved through parallelization of data analysis. VAP currently supports datasets in BedGraph and WIG format, but will also eventually support BigWig and BAM formats. Based on its overall efficiency, VAP can run either on a laptop computer or on a server.

### Usage

VAP functionalities are available both through a user-friendly interface and through the command line. The interface is written in Java (requiring version 7), while the core of VAP is written in C++. The interface guides the user to create a parameter file (plain text format), which is automatically sent to the core executable to analyze the data and generate graphical representations of the aggregate profiles (with the possibility of combining multiple datasets, reference groups and orientation subgroups on the same graph). A preexisting parameter file can also be loaded by the interface. The results are output in a tab-delimited text file that can be used to re-create graphs using external software such as GraphPad or the Libre/Open Office and Microsoft Office macro included in provided spreadsheets to produce publication-ready vectorial graphs. The user can decide to add a prefix to each output file to facilitate the exploration of the parameters.

**Figure 5.**
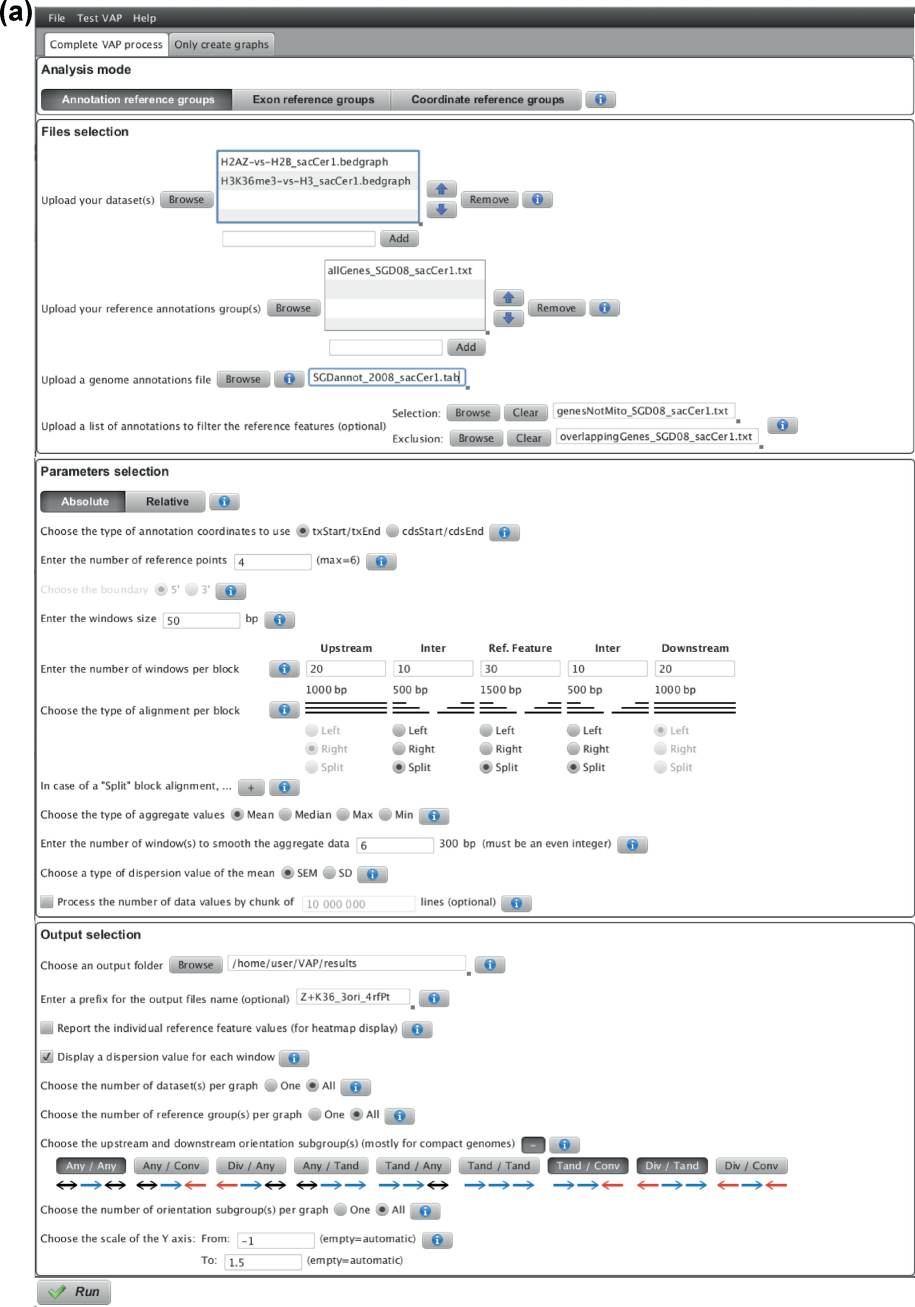
Annotated screenshot. (**a**) Interface filled with the parameters used to generate Figure 2e. Using the annotation mode, the H2A.Z and H3K36me3 datasets were analyzed over a reference group containing all the yeast genes (therefore using the corresponding genome annotations file from the Saccharomyces Genome Database (41)) with filters selecting the non-mitochondrial genes and excluding the overlapping genes, where all files used the sacCer1 assembly. The aggregate profile was generated using the absolute method with four reference points to isolate the signal over the reference features as well as their complete flanking intergenic regions with a resolution of 50 bp. Using four reference points creates five blocks corresponding, respectively, to the upstream annotations, upstream intergenic regions, reference features (each gene from the reference group), downstream intergenic regions and downstream annotations. The number of 50 bp windows per block was chosen such that the aggregate profiles cover up to 1 kb, 500 bp, 1.5 kb, 500 bp and 1 kb for each block, respectively. The first and last blocks are always aligned respectively to the right and to the left, while the other blocks were split (in the middle by default). This means that a gene (reference feature) of 1 kb will contribute to 20 of the 30 windows of the third block (first and last 10 windows of this block), while a 2 kb gene will contribute to all the windows of this block (the middle 500 bp being ignored). The aggregate value is the mean, a smoothing of six windows is applied on the aggregate values, and the SEM is calculated. The aggregate profile of all the datasets and orientation subgroups were combined on the same image, showing the profile of all genes as well as two orientation subgroups of genes with a predetermined Y-axis scaling.

The main interface presents the three analysis modes (‘Annotation’, ‘Exon’ and ‘Coordinates’) along with their relevant parameters (Figure 5a). Moreover, the user can chose from the ‘Absolute’ or the ‘Relative’ method to analyze the data. Relevant files and folders can be uploaded by ‘Drag and drop’ or by using the ‘Browse’ function (allowing multiple selections at a time). Alternatively, the full path can be pasted into the appropriate box, followed by clicking the ‘Add’ button. While running, a progression bar indicates to the user the different steps, and logfiles with more details are generated. By hitting the ‘Run’ button, the interface detects the Operating System (OS) configuration and decompresses the appropriate executable (compiled with g++ 4.2) in the output directory selected by the user. To allow users having to compile the core code on their computer to still benefit from the interface, VAP first looks for the presence of a binary named ‘vap_native’ in the output directory and will use it rather than one executable from the package. Sample data are also packaged within the interface, allowing the user to test the versatile functionalities of VAP.

As mentioned above, it is also possible to directly use VAP from the command line. In this case, a parameter file (modified or not from a file created by the interface, or manually created) is used as an argument to the core executable: vap_core –p paramFile. To generate graphical representations from the files produced using the command line, it is also possible to use the command line or a specific tab of the interface (Figure 5a). VAP is under active development and more features will be added in the near future.

## CONCLUSION

VAP is a user-friendly stand-alone tool to flexibly generate aggregate or individual profiles of large genomic datasets such as ChIP and transcriptomic data over groups of reference features (genes, annotations, regions) of interest. Both the absolute and relative methods are offered, and as demonstrated, the choice of methodology is important to avoid incorrect interpretation of the results. VAP also permits up to six reference points to delimit the sections of interest in order to avoid contamination of the signal from adjacent features. In the ‘Annotation’ and ‘Exon’ analysis modes, the reference groups are simply composed of unique names linked to a genome annotation file, while users can directly provide the coordinates of the reference points in the ‘Coordinate’ mode. Statistical measures, which can be displayed on the aggregate curves, facilitate comparisons between groups or datasets. Moreover, subgrouping based on the orientation of the flanking annotations is particularly useful for compact genomes. VAP targets both biologists, through an intuitive interface, and bioinformaticians, through command line interactivity. Being highly efficient and given its ability to limit its memory footprint, VAP is designed to run on laptop computers, but it can also be compiled and run on a server.

## AVAILABILITY

VAP is open source, published under the GNU General Public License v3. The official VAP website (http://lab-jacques.recherche.usherbrooke.ca/vap) contains complete documentation as well as links to download the packaged jar files, which contain the executables (32 and 64 bit architecture) for the supported OS (Linux, Mac OS X and Windows). A Bitbucket account (labjacquespe/vap) contains the source code of both the C++ (vap_core) and Java (vap_interface) modules with the corresponding makefiles and dependencies, as well as example input and output files.

## SUPPLEMENTARY DATA

Supplementary Data are available at NAR Online.

Supplementary Data
