# Supplementary material for: VAP: a versatile aggregate profiler for efficient genome-wide data representation and discovery
Source: Nucleic Acids Res. 2014 Apr 21;42(Web Server issue):W485–93. doi: 10.1093/nar/gku302 (PMC4086060; doi:10.1093/nar/gku302)
Supplement: Supplementary Data [file supp_42_W1_W485__index.html]

Supplementary Data 

# VAP: a versatile aggregate profiler for efficient genome-wide data representation and discovery

## Supplementary Data

**Files in this Data Supplement:**

- SUPPLEMENTARY DATA
