# Supplementary material for: VAP: a versatile aggregate profiler for efficient genome-wide data representation and discovery
Source: Nucleic Acids Res. 2014 Apr 21;42(Web Server issue):W485–93. doi: 10.1093/nar/gku302 (PMC4086060; doi:10.1093/nar/gku302)
Supplement: Supplementary Data [file supp_gku302_nar-00329-web-b-2014-File007.pdf]

VAP: a versatile aggregate profiler for efficient genome-wide data representation and discovery

Coulombe Charles[1], Poitras Christian[2], Nordell-Markovits Alexei[1,3], Brunelle Mylène[3], Lavoie Marc-André[3], Robert François[2,4,*] and Jacques Pierre-Étienne [1,3,5,*]

[1] Département d'informatique, Faculté des sciences, Université de Sherbrooke, Sherbrooke, Québec, J1K 2R1, Canada
[2] Institut de recherches cliniques de Montréal, Montréal, Québec, H2W 1R7, Canada
[3] Département de biologie, Faculté des sciences, Université de Sherbrooke, Sherbrooke, Québec, J1K 2R1, Canada
[4] Département de médecine, Faculté de médecine, Université de Montréal, Montréal, Québec, Canada
[5] Centre de recherche du Centre hospitalier universitaire de Sherbrooke, Sherbrooke, Québec, J1H 5N4, Canada

* To whom correspondence should be addressed. Tel: 1-819-821-8000#65914; Fax: 1-819-821-8049; Email: Pierre-Etienne.Jacques@USherbrooke.ca
Correspondence may also be addressed to Francois.Robert@ircm.qc.ca

SUPPLEMENTARY DATA

This file contains 2 supplementary figures.

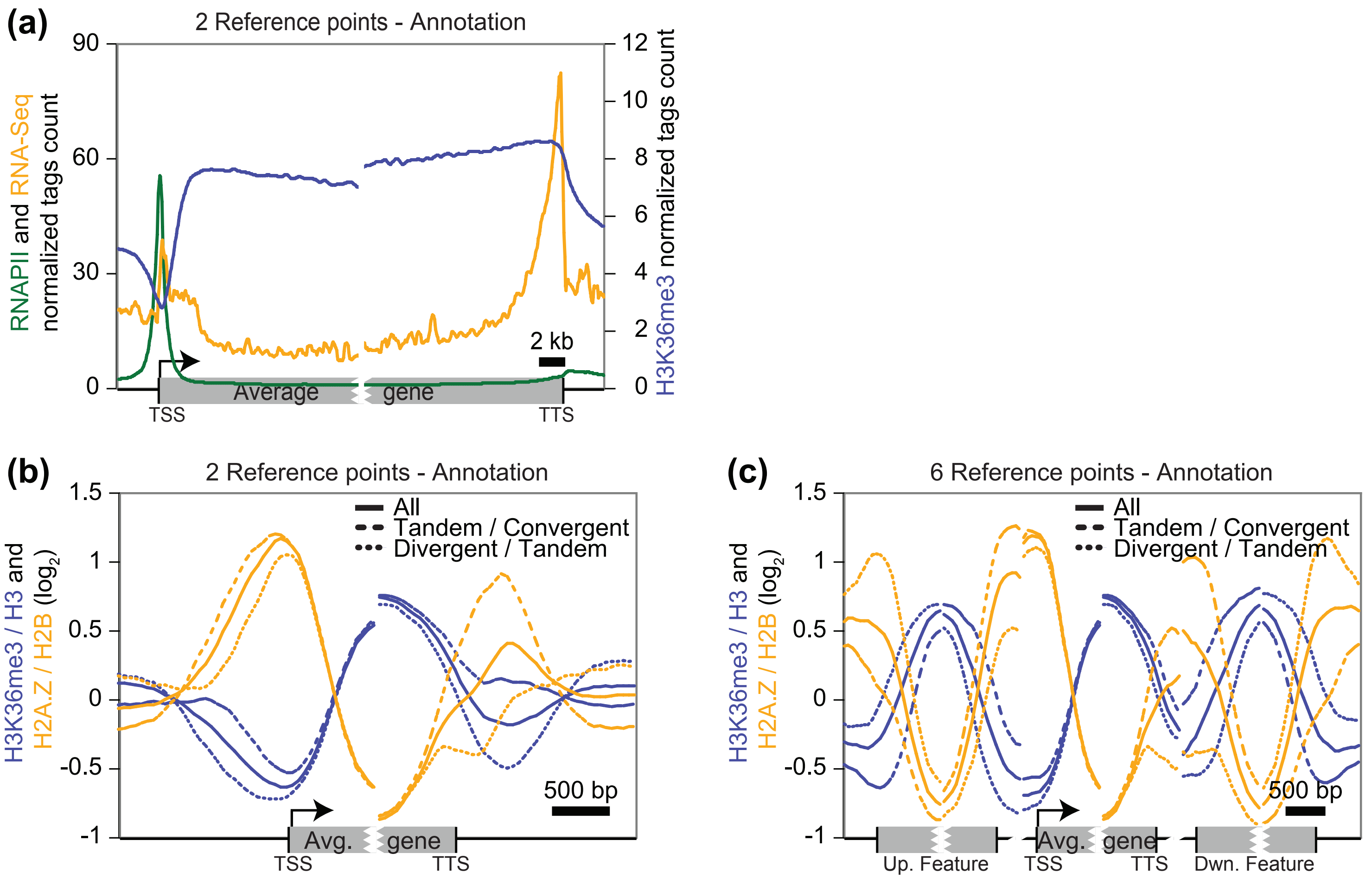

Figure S1. Supporting the material of Figure 2.

(a) As in Figure 2d but using two reference points in the annotation mode, and including a RNA-Seq dataset from ENCODE (32) in HeLa cells (same cells than Figure 2d).

(b) As in Figure 2e but using two reference points.

(c) As in Figure 2e but using six reference points.

(a)

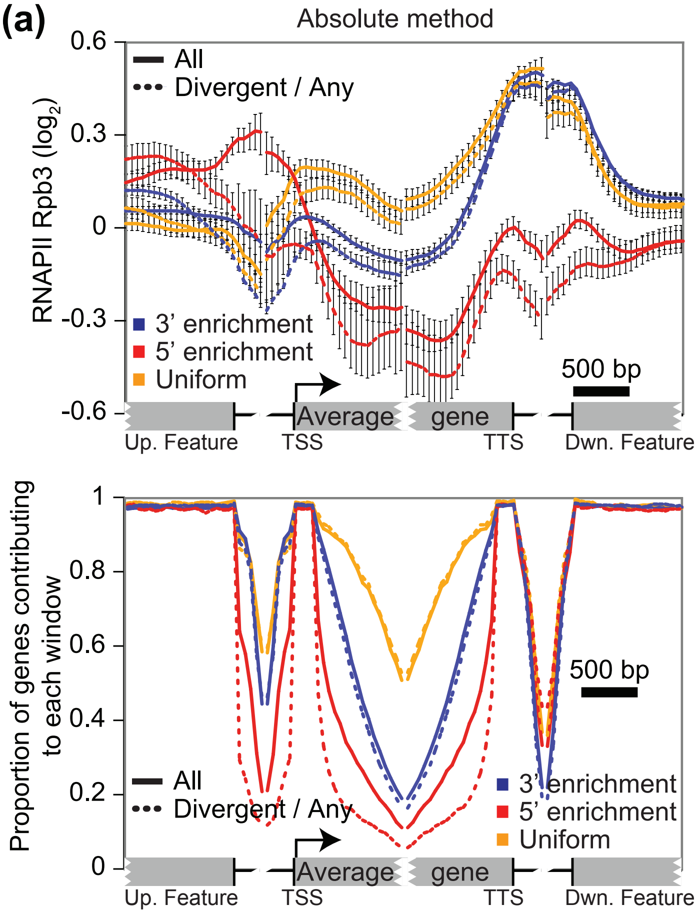

Figure S2. Supporting the material of Figure 3.
(a) Aggregate (top) and proportion (bottom) profiles showing the RNAPII subunit Rpb3 dataset (25) on the same groups of genes than Figure 3d, including the SEM.
